# Enhancing Violence Risk Assessment and Management with eHARM and AIS: Advancements in Digital Tools in Forensic Psychiatry

**DOI:** 10.1192/j.eurpsy.2026.12065

**Published:** 2026-07-31

**Authors:** G. Chaimowitz, M. Mamak, G. Behravan

**Affiliations:** St. Joseph’s Healthcare Hamilton, Hamilton, Canada

## Abstract

**Introduction:**

Violence risk assessment plays a crucial role in forensic psychiatry, as the accurate evaluation and management of aggressive behaviors are essential in clinical practice. The Hamilton Anatomy of Risk Management (eHARM) and the Aggressive Incident Scale (AIS) are widely used tools designed to assess and manage the risk of violence in psychiatric and forensic settings. Transitioning to cloud-based platforms has helped improve their utility, accessibility, and real-time effectiveness in clinical environments.

**Objectives:**

This presentation aims to explore the recent advancements in the eHARM and AIS, with a focus on their transition to digital platforms. Specifically, the presentation will highlight the benefits of these updates, their impact on clinical practice, and their potential to enhance patient care, improve safety, and routine assessments.

**Methods:**

The eHARM, now available as a cloud-based platform, allows for real-time data collection, dynamic risk tracking, and a more personalized approach to patient risk assessment. The Aggressive Incident Scale (AIS), used to document and assess violent behaviors, has also been adapted for cloud-based monitoring, and enables more efficient documentation, instant updates on behavior trends, and timely interventions. The transition to cloud technology ensures that both eHARM and AIS can be easily integrated into diverse clinical settings, including remote or underserved areas, and which further extends their reach and utility.

**Results:**

The cloud-based versions of eHARM and AIS have shown promising improvements in several areas. These tools now allow for real-time tracking and assessment of both individual and population-level risk factors, and facilitate more timely and informed interventions. The transition to digital platforms has also improved the communication of risk-related data among clinicians, which enhances collaboration and decision-making processes. Moreover, the adaptability of these tools across different clinical settings has demonstrated their effectiveness in diverse environments, and helped improve the consistency and quality of risk management practices. Additionally, the updates to AIS, including the integration of problematic sexual behaviors and bullying, have helped expand its scope, which enables clinicians to better monitor and respond to a broader range of aggressive behaviors.

**Conclusions:**

The digital transformation of eHARM and AIS represents a significant advancement in violence risk assessment and management in forensic psychiatry. By leveraging cloud-based technology, these tools offer increased flexibility, real-time monitoring, and enhanced decision-making capabilities. The integration of these tools into clinical practice represents a step forward in the broader goal of improving clinical outcomes and reducing the impact of violence and aggression in psychiatric settings.

**Disclosure of Interest:**

None Declared

